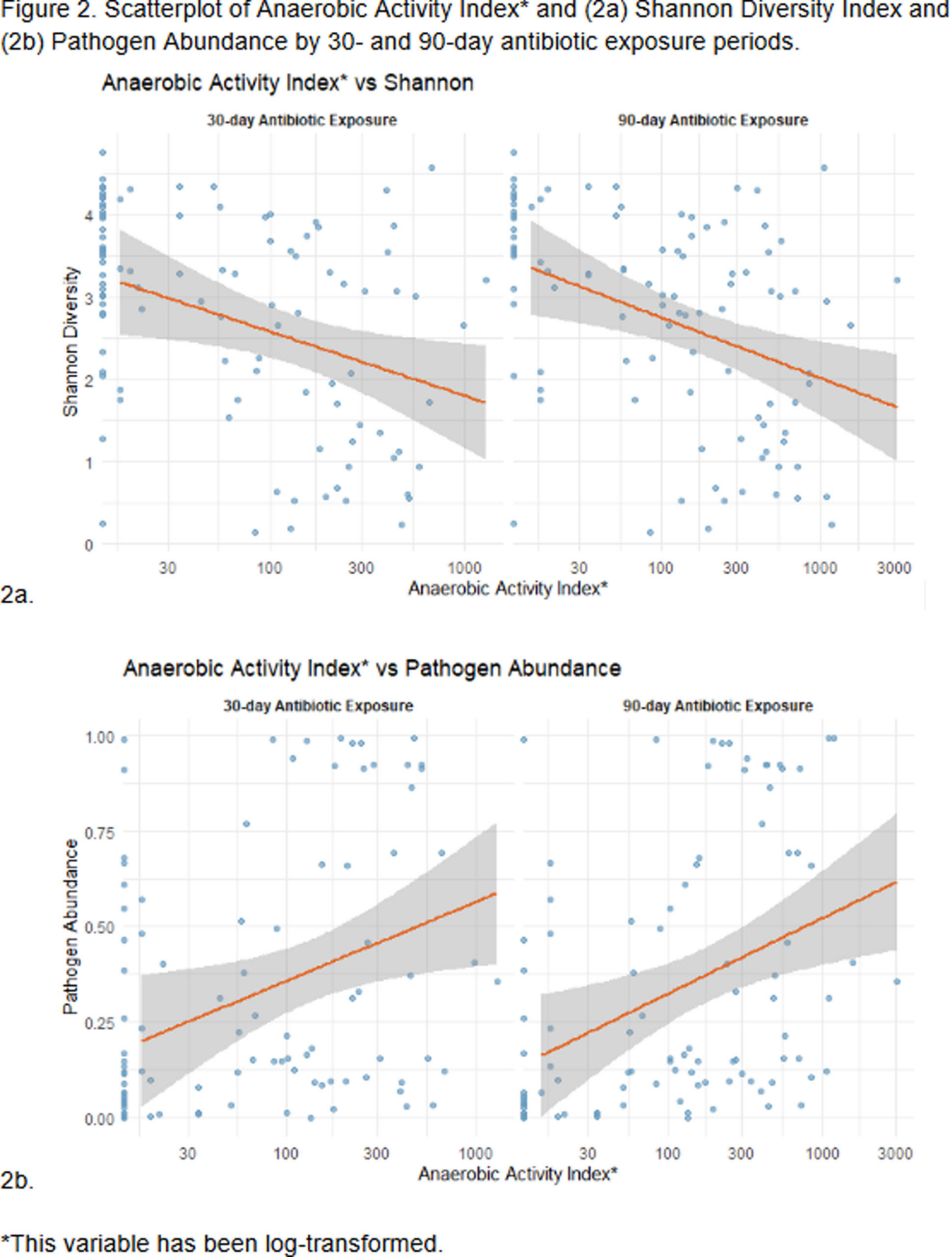# 9 Utilization of Analytics Software and Whole Genome Sequencing to Identify an Environmental Legionella Cluster

**DOI:** 10.1017/ash.2026.10463

**Published:** 2026-06-23

**Authors:** Lindsey Walker, Deepti Suchindran, Ashton Self, Brianna Delph, Dylan Koundakjian, Ahmed Babiker, Beth Coughlin, Annie Taing, Sarah Lohsen, Sarah Satola, Scott Fridkin, Michael Woodworth

**Affiliations:** 1 Emory University; 2 Emory School of Medicine; 3 Emory University School of Medicine, Division of Infectious Diseases, Department of Medicine, Atlanta GA; 4 Beth Israel Deaconess Medical Center; 5 Rush Medical Center; 6 Emory University School of Medicine; 7 Emory Healthcare and Emory University; 8 Division of Infectious Diseases, Department of Medicine, Emory University, Atlanta, GA

## Abstract

**Background:** Microbiome disruption is linked to risk of mortality and infection. Measuring the impact of patient-level antibiotic exposure to microbiome disruption is needed to inform stewardship and microbiome restoration therapy efforts. The National Healthcare Safety Network (NHSN) standard, days of therapy (DOT), widely used to capture antibiotic use, does not account for spectrum of activity. Other metrics better account for spectrum, including antibiotic spectrum coverage (ASC), antibiotic spectrum index (ASI), and anaerobic activity index (AAI) – and with NHSN antibiotic groups (Clostridioides difficile infection [CDI] high risk, and broad-spectrum hospital-onset infections [BSHO]). We compared DOT with spectrum-weighted metrics to determine which best track patient-level microbiome disruption. **Method:** We retrospectively analyzed peri-rectal swab metagenomic data and antibiotic use for acute and long-term care facility patients. Shannon diversity index, pathogen abundance, and butyrate-producing bacterial abundance were calculated for each participant. Antibiotic exposure within 30 and 90 days prior to sampling were summarized six ways: crude duration – DOT for all antibiotics, NHSN-CDI antibiotics, and NHSN-BSHO antibiotics (all unweighted) and three weighted DOT values (sum of weights per day for each ASC, ASI, and AAI) across all days in the exposure window, accounting for antibiotic spectrum. Spearman correlations were calculated among all six antibiotic metrics and between the metrics and microbiome disruption features. **Result:** One hundred participants were included with median 30-day and 90-day DOT of 16 (IQR:6-31) and 27 (IQR: 13-64), respectively. Weighted metrics (ASC, ASI, AAI) demonstrated strong correlations with each other (Figure 1, darker orange). Crude duration metrics (DOT, CDI, BSHO) were only moderately correlated with weighted metrics (Figure 1, lighter orange). Shannon diversity was correlated with all exposure metrics except the NHSN CDI high-risk group, and AAI had the strongest association (30-day r=-0.41, p **Conclusion:** Patient-specific weighted DOT of antibiotic exposure, in particular the AAI weights, correlates with gut microbiome disruption well. Interestingly, the NHSN high-CDI-risk antibiotic group did not. Patient-level 30-day exposure had similar observed correlations as 90-day exposure windows and may be a practical window to measure microbiome antibiotic effects. Further study is needed to test causality of identified associations, the influence of absolute vs relative abundance measures, and best ways to incorporate these measures into stewardship efforts and identification of microbiota therapy candidates.

**Figure:**
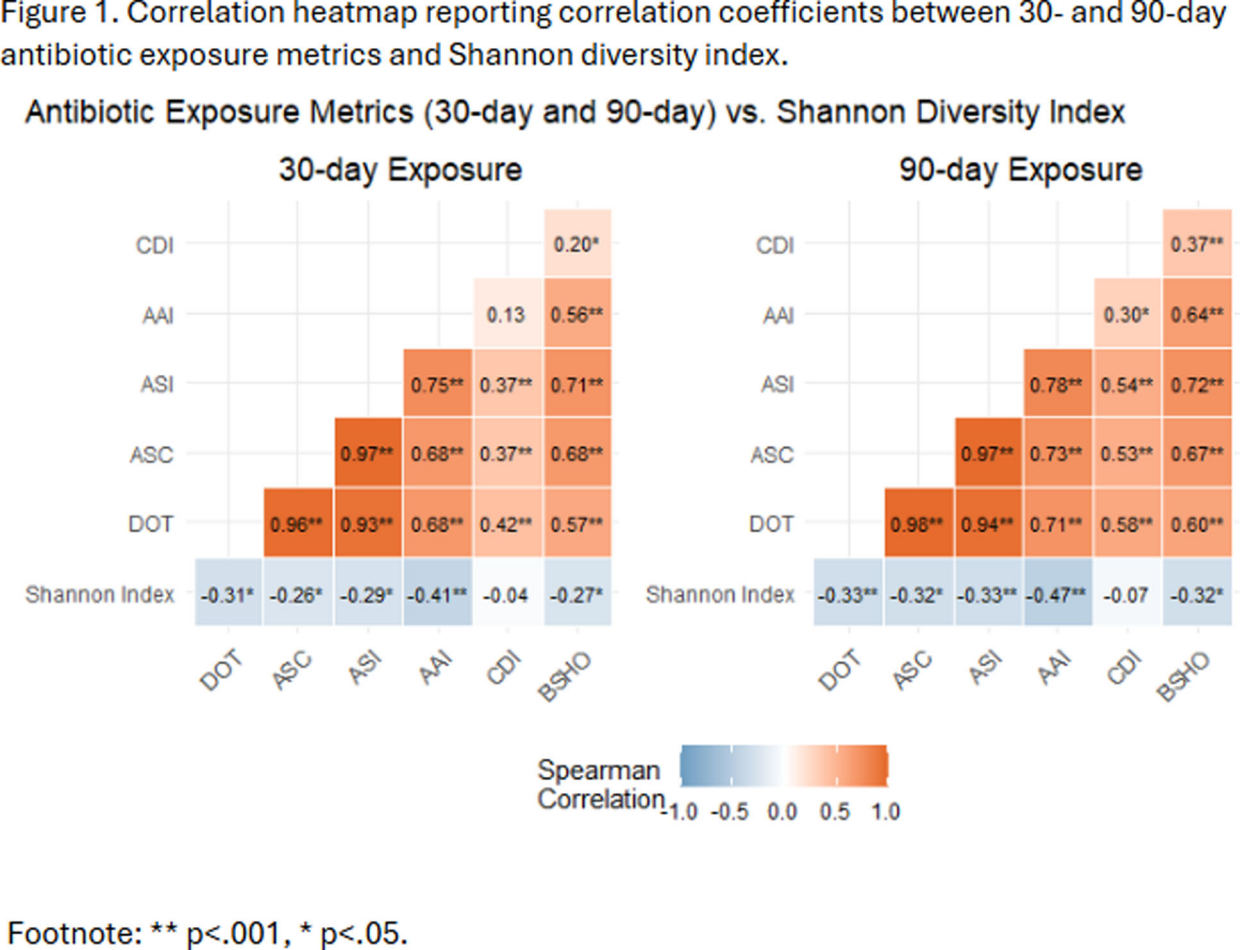

**Figure:**